# Fractionation, Structural Characterization and In Vitro α-Amylase and α-Glucosidase Inhibitory Activities of Polysaccharides Derived from *Lycium barbarum* L.

**DOI:** 10.3390/molecules31213839

**Published:** 2026-10-23

**Authors:** Xuan Zhou, Yanyan Liu, Ting Huang, Linyuan Duan, Yuekun Li

**Affiliations:** 1 National Wolfberry Engineering Research Center, Wolfberry Science Research Institute, Ningxia Academy of Agriculture and Forestry Sciences, Yinchuan 750002, China; 2 School of Pharmacy, Gansu University of Chinese Medicine, Lanzhou 730000, China

**Keywords:** *Lycium barbarum* polysaccharides, in vitro α-amylase inhibition, α-glucosidase inhibition, structural characterization, galacturonic acid, structure–activity relationship

## Abstract

Polysaccharides have attracted extensive attention due to their pharmacological activities, such as regulating depression, antitumor, hypoglycemic and lipid-lowering effects, and regulating intestinal flora. In this study, four crude polysaccharide fractions, designated LICP007, LICP008, LICP009 and LICP010, were prepared from *Lycium barbarum* L. Based on the in vitro α-amylase and α-glucosidase inhibitory assays, LICP007, which showed the strongest activity among the four crude polysaccharides (inhibition rates of 61.42 ± 0.29% against α-amylase and 40.69 ± 0.35% against α-glucosidase at 50 mg/mL), was selected for further separation and purification, and one neutral polysaccharide (LICP007-N1) and two acidic polysaccharides (LICP007-S2 and LICP007-S5) were obtained by DEAE-52 cellulose anion-exchange and Sephadex G-200 gel permeation chromatography. Among them, LICP007-S5 showed the best inhibitory activity against α-amylase and α-glucosidase, with inhibition rates of 98.38% and 43.45% at 20 mg/mL, respectively. Physicochemical characterization showed that LICP007-S5 differed markedly from LICP007-N1 and LICP007-S2 in molecular weight distribution, monosaccharide composition and morphology. Structural analysis indicated that LICP007-S5 is an acidic heteropolysaccharide rich in galacturonic acid (27.5 mol%); atomic force microscopy (AFM) and Congo red test results suggest that it may adopt an ordered, possibly triple-helical conformation, although this requires further verification. These findings provide comparative information on the structure and biological activity of different *Lycium barbarum* polysaccharides and highlight their potential as natural α-amylase and α-glucosidase inhibitors for the dietary management of type 2 diabetes in functional foods.

## 1. Introduction

Diabetes mellitus (DM) is a common clinical metabolic disease that is caused by a deficiency in insulin secretion or impairment of its biological effects. Its development is closely associated with both genetic and environmental factors. The prevalence and incidence of DM have increased in recent decades, and approximately 10% of the global population already has type 2 diabetes or is likely to develop it [1]. Long-term hyperglycemia directly damages various tissues and organs, including the eyes, kidneys and heart; in severe cases, it can lead to blindness or amputation, seriously compromising patients’ health and quality of life [2]. Currently, the most effective and widely used chemical drugs in clinical practice include sulfonylureas, thiazolidinediones, α-glucosidase inhibitors, biguanides, DPP-4 inhibitors and GLP-1 analogs. As the treatment of type 2 diabetes requires long-term medication, side effects and toxicity cannot be ignored [3]. Therefore, one of the main directions of scientific research is the development of safer and more effective drugs for the treatment of type 2 diabetes without toxic side effects. Meanwhile, polysaccharides derived from natural resources have attracted worldwide attention owing to their diverse bioactivities, including antioxidant, immunomodulatory, antidiabetic, and antitumor activities [4].

*Lycium barbarum* L. is a traditional food in China and other Asian countries. As a multipurpose medicinal and edible plant, *Lycium barbarum* L. contains abundant natural active components. Numerous medical studies have demonstrated that *L. barbarum* confers a variety of medicinal properties, including antiaging effects [5], neuroprotection [6], improved immune function [7], hypoglycemic effects [8], hypolipidemic effects [9], antitumor effects [10] and antioxidation [10]. It can also alleviate visual fatigue and myocardial injury [11], reduce radiation-induced damage and regulate the intestinal flora [12], and act as an auxiliary treatment for chemical liver injury. *Lycium barbarum* polysaccharides (LBPs) are widely regarded as the major active components responsible for these physiological activities. LBPs are water-soluble heteropolysaccharides with a molecular weight of 10–2300 kDa [13], accounting for 5–8% of dried fruits, and comprise predominantly glucose, arabinose, mannose, xylose, rhamnose, and fructose, as well as various polysaccharide complexes. An increasing number of studies have demonstrated that LBPs confer hypoglycemic effects [14,15], although the structural basis underlying these effects remains unclear.

Therefore, based on previous studies, this study screened the components of LBPs for in vitro α-amylase and α-glucosidase inhibitory activity and further separated and purified the most active fraction to determine whether any components of *L. barbarum* possess strong in vitro enzyme inhibitory activity and to clarify the material basis underlying the potential hypoglycemic effects of LBPs. Existing studies have primarily compared crude LBPs from different cultivars, regions and processing methods [16], or characterized the structure–function properties of LBPs using spectroscopic and enzymatic fingerprinting approaches [17,18]; however, few studies have linked the fine structural features of individually purified LBP fractions to their in vitro α-amylase and α-glucosidase inhibitory activities. Compared with these earlier studies, the present work links fractionation, structural characterization and enzyme-inhibitory activity evaluation in a single workflow: crude LBP fractions of different molecular weights were screened for α-amylase and α-glucosidase inhibitory activity, the most active fraction was further purified by ion-exchange and gel-filtration chromatography, and the purified subfractions were comprehensively characterized, so that the fractionation-dependent structural features of LBPs could be directly related to their enzyme-inhibitory activities. The results are expected to clarify the differences in the structure and biological activity of different polysaccharide fractions and provide potential natural α-amylase and α-glucosidase inhibitors for the pharmaceutical and food industries.

## 2. Results and Discussion

### 2.1. Separation and Purification of Crude Polysaccharides

**Figure 1:**
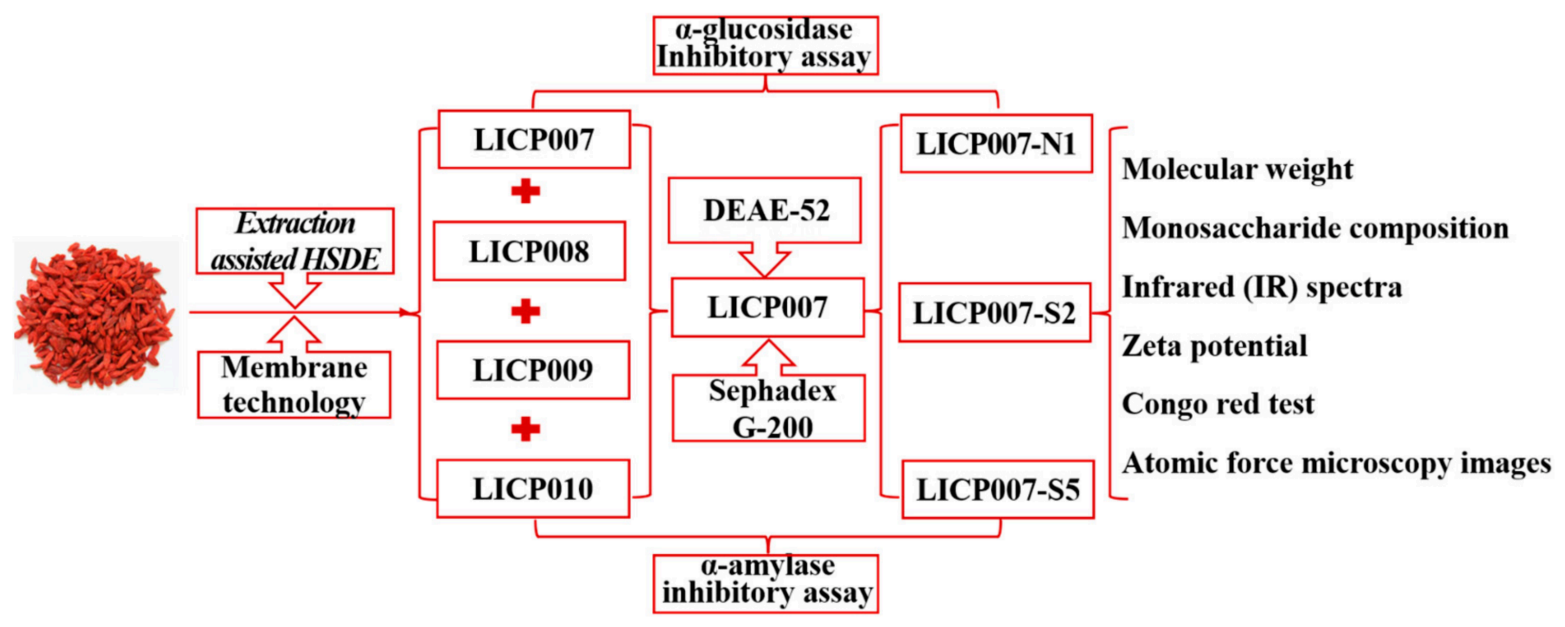
A scheme for extraction and fractionation of polysaccharides from *Lycium barbarum* L.

Changes in the molecular weight of polysaccharides may lead to different pharmacological effects. Hierarchical alcohol precipitation is the most popular method for the separation of polysaccharides. However, dehydration of polysaccharides in ethanol solution can transform their conformation through intramolecular hydrogen-bond reassembly, which complicates structure–activity studies [19]. In this work, integrated tandem hybrid membrane technology, which was developed by our team, was used to separate polysaccharides from *L. barbarum* into fractions designated LICP007, LICP008, LICP009, and LICP010 (Figure 1). Compared with hierarchical alcohol precipitation, this technology is a size-exclusion-based physical process that avoids structural changes to the polysaccharides during separation [19]. As shown in Table 1, LICP007 gave the highest yield (4.10%), whereas the other three fractions gave yields of 2.16–2.74%. The results of the iodine, Fehling’s, biuret, and ferric chloride tests indicated that all four fractions did not contain starch or free monosaccharides but contained proteins and polyphenols. This indicated that both deproteinization and depigmentation were necessary before further purification. All four fractions contained uronic acid; LICP007 showed the highest uronic acid (18.2%) and total carbohydrate (65.3%) contents, and LICP010 the lowest (Table 1). Uronic acids and total carbohydrates are the major contributors to the biological activity of polysaccharides [20].

### 2.2. Inhibitory Effects on α-Amylase Activity

**Figure 2:**
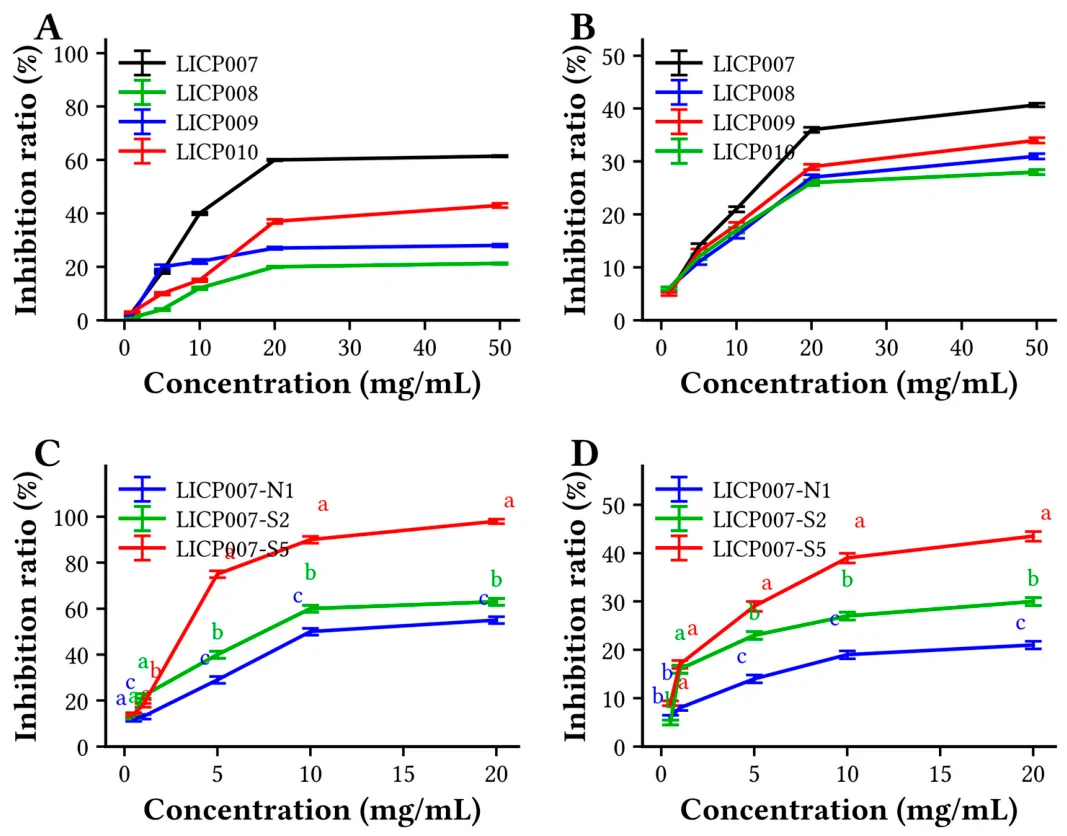
Inhibitory effects of the crude polysaccharides (LICP007, LICP008, LICP009 and LICP010) on (**A**) α-amylase and (**B**) α-glucosidase activity at concentrations of 1–50 mg/mL; inhibitory effects of the purified fractions (LICP007-N1, LICP007-S2 and LICP007-S5) on (**C**) α-amylase and (**D**) α-glucosidase activity at concentrations of 0.5–20 mg/mL. Data are expressed as the mean ± SD (*n* = 3). Different lowercase letters above the data points in (**C**,**D**) indicate significant differences among the three fractions at the same concentration (*p* < 0.05, Tukey’s HSD test).

As shown in Figure 2A, LICP007 showed the highest inhibitory activity against α-amylase, with an inhibition rate of 61.42 ± 0.29% at 50 mg/mL, whereas LICP008 showed the weakest activity (21.29 ± 0.22%). This indicated that LICP007 was the fraction with the strongest in vitro enzyme inhibitory activity and was therefore selected for further separation and purification. The inhibitory effects of LICP007-N1, LICP007-S2 and LICP007-S5 on α-amylase were investigated, and all samples exhibited in vitro α-amylase inhibitory activity. The inhibition rates increased with sample concentration, showing a dose-dependent effect over 0.5–20 mg/mL. Moreover, LICP007-S5 showed a stronger inhibitory effect on α-amylase than LICP007-N1 and LICP007-S2. As shown in Figure 2C, the inhibition ratio of LICP007-S5 increased from 13.41% at 0.5 mg/mL to 98.38% at 20 mg/mL, the strongest α-amylase inhibition among the three fractions. The IC50 values of LICP007-N1, LICP007-S2 and LICP007-S5 against α-amylase were 10.12, 7.46 and 3.28 mg/mL, respectively. Statistical analysis confirmed that the differences among the three fractions at each concentration were significant (*p* < 0.05, Tukey’s HSD test), except at 0.5 mg/mL, where no significant difference was observed, and the corresponding significance letters are marked in Figure 2C. Under the same assay conditions, the positive control acarbose showed strong, concentration-dependent inhibition of α-amylase, confirming the validity of the assay system.

### 2.3. Inhibitory Effects on α-Glucosidase Activity

As shown in Figure 2B, all four crude polysaccharides inhibited α-glucosidase in a concentration-dependent manner over 1–50 mg/mL. In particular, LICP007 showed a markedly stronger inhibitory effect than the other three fractions, with an inhibition rate of 40.69 ± 0.35% at 50 mg/mL. LICP010 showed the weakest α-glucosidase inhibitory activity. As shown in Figure 2D, LICP007-S5 showed a higher inhibitory effect on α-glucosidase than LICP007-N1 and LICP007-S2. At 20 mg/mL, LICP007-S5 showed the highest inhibition rate (43.45%), followed by LICP007-S2 and LICP007-N1 (21.0%). The IC50 values of LICP007-N1, LICP007-S2 and LICP007-S5 against α-glucosidase were all higher than 20 mg/mL, since none of the fractions reached 50% inhibition within the tested concentration range. The differences among the three fractions were confirmed to be statistically significant (*p* < 0.05, Tukey’s HSD test), and the corresponding significance letters are marked in Figure 2D. Together, these results indicated that LICP007-S5 has greater potential as an in vitro α-glucosidase inhibitor than the other fractions, which may be associated with differences in their structural characteristics, including monosaccharide compositions, molecular weight, glycosyl linkage types and molecular conformation [21]. Similarly, under the same assay conditions, the positive control acarbose inhibited α-glucosidase in a concentration-dependent manner, confirming the validity of the assay system.

### 2.4. Purification of the Polysaccharide Fraction LICP007

**Figure 3:**
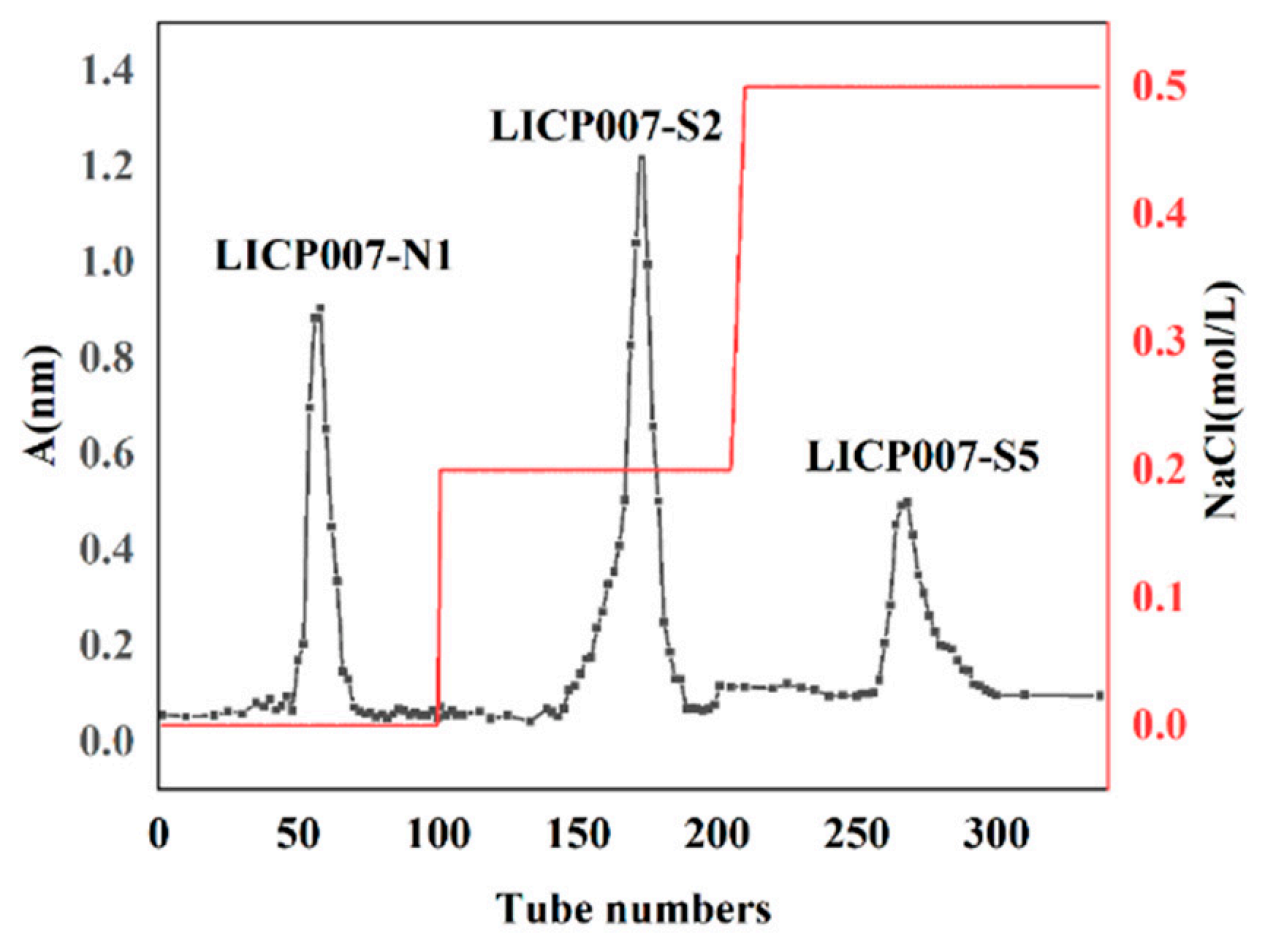
Elution curve of LICP007 on the DEAE-52 cellulose column, showing the LICP007-N1, LICP007-S2 and LICP007-S5 fractions.

After deproteinization, decolorization, dialysis, and lyophilization, LICP007 was then dissolved in distilled water, loaded onto a DEAE-52 cellulose column (5.5 cm × 60 cm), and eluted stepwise with distilled water, 0.2 mol/L NaCl, and 0.5 mol/L NaCl solutions. The elution curve is illustrated in Figure 3. Three polysaccharide fractions were obtained, with yields of 12.34%, 11.44% and 18.09% (relative to the loaded LICP007) for the water-, 0.2 mol/L NaCl- and 0.5 mol/L NaCl-eluted fractions, respectively. After purification by a Sephadex G-200 column, three purified polysaccharides, LICP007-N1, LICP007-S2 and LICP007-S5, were obtained (0.1214 g, 0.2499 g and 0.2618 g, corresponding to overall yields of 4.44%, 9.14% and 9.58% relative to the LICP007 subjected to fractionation (2.73 g), respectively).

### 2.5. Structural Characterization

#### 2.5.1. Molecular Weight Distribution

**Figure 4:**
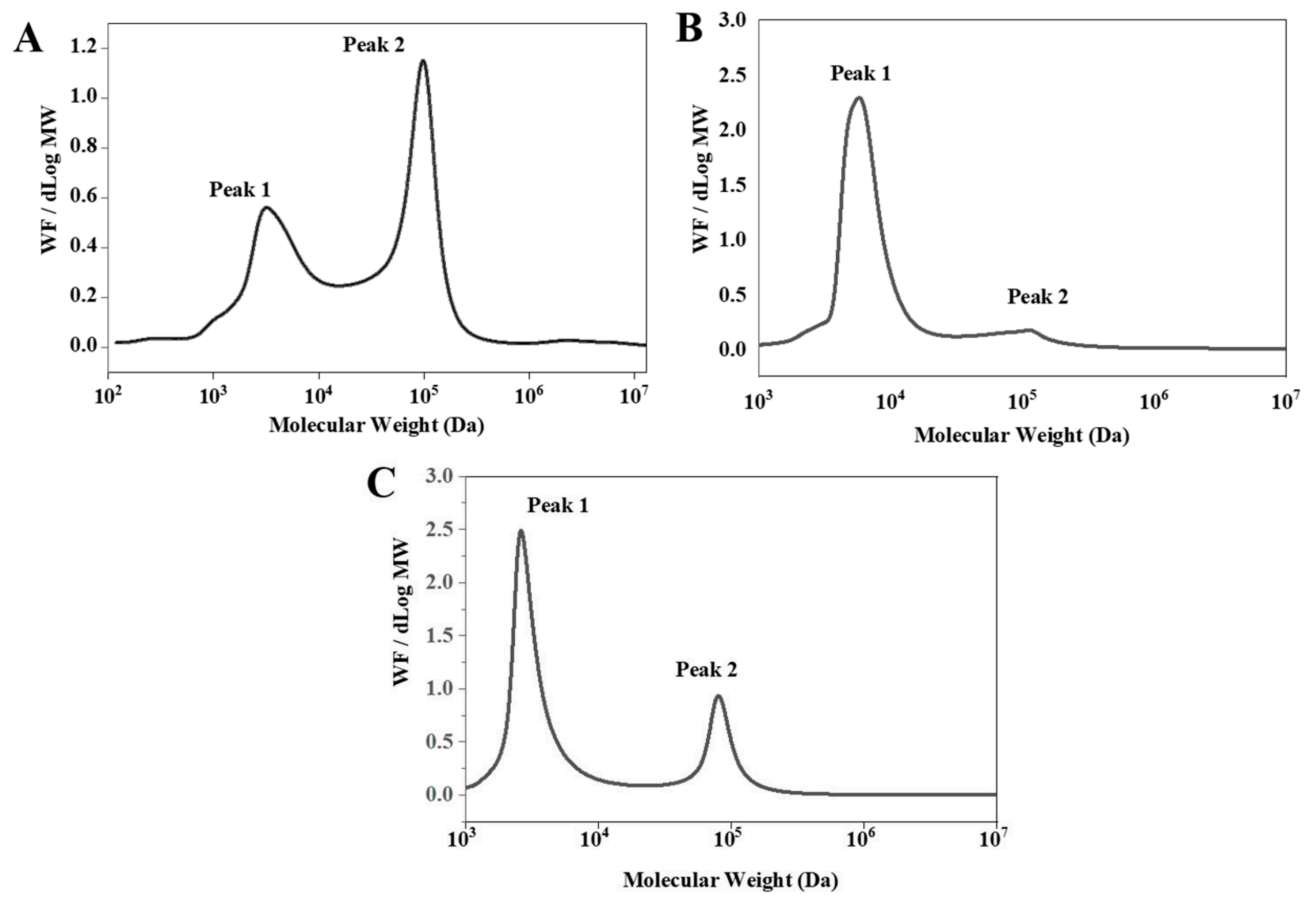
HPSEC-MALLS-RID chromatograms of LICP007-N1 (**A**), LICP007-S2 (**B**), and LICP007-S5 (**C**).

Polysaccharides are mixtures of chains with different lengths, and their measured MWs are averages over these homologs. High-performance size-exclusion chromatography coupled with multi-angle laser light scattering and a refractive index detector (HPSEC-MALLS-RID) can provide the weight-average molecular weight (*M*_w_), number-average molecular weight (*_M_*_n_), z-average molecular weight (*_M_*_z_) and polydispersity index (PDI) values (*_M_*_w_/*_M_*_n_) of three polysaccharides (LICP007-N1, LICP007-S2 and LICP007-S5), as well as the radius of gyration (*R*_g_), which estimates the approximate molecular size, and the specific volume for gyration (*SV*_g_), which reflects molecular compactness. As shown in Table 2 and Figure 4, LICP007-N1 exhibited two major peaks with *M*_w_ of 4.229 × 10^3^ and 1.042 × 10^5^ g/mol and PDI values of 1.317 and 2.309, respectively; its *R*_g_ values spanned 4.85–82.7 nm, and the *SV*_g_ values calculated by the equation of You and Lim varied between 0.144 and 12.434 cm^3^/g [22], indicating that LICP007-N1 was highly heterogeneous in molecular size distribution. LICP007-S2 showed a single, broad *M*_w_ distribution (*_M_*_w_ 5.368 × 10^4^ g/mol, PDI 6.111), characteristic of a polydisperse heteropolysaccharide (Figure 4B). Similarly, LICP007-S5 showed two peaks (Figure 4C) with *M*_w_ of 9.202 × 10^3^ and 1.031 × 10^5^ g/mol, respectively. Meanwhile, the PDI values of peaks 1–2 in LICP007-S5 were 2.002 and 1.31, indicating that peak 2 showed a relatively narrow molecular-weight distribution, whereas peak 1 was broader (PDI = 2.002), and neither peak should be regarded as strictly monodisperse. LICP007-S5 was fractionated by charge (anion-exchange) rather than by molecular size and may therefore contain more than one molecular-weight population; chromatographic peak homogeneity does not necessarily imply chemical homogeneity [23]. It is noteworthy that, for both LICP007-N1 and LICP007-S5, the lower-molecular-weight peak exhibited the larger *R*_g_ value (Table 2). This inverse *R*_g_–*M*_w_ relationship may reflect chain aggregation or a more extended conformation of the low-molecular-weight population in the eluent; moreover, *R*_g_ values below approximately 10 nm approach the lower size limit that can be reliably determined by MALLS and should therefore be interpreted with caution. These results showed that the three fractions differed markedly in molecular weight, *M*_w_ distribution, particle size, and molecular compactness—not only between the neutral and acidic polysaccharides, but also between the two acidic polysaccharides. The relationship between these differences and enzyme inhibitory activity requires further study.

#### 2.5.2. Monosaccharide Composition

The monosaccharide compositions of LICP007-N1, LICP007-S2 and LICP007-S5 were analyzed by ion chromatography, as shown in Table 3. All three fractions were heteropolysaccharides. LICP007-N1 and LICP007-S2 were both dominated by arabinose, galactose and glucose, whereas LICP007-S5 was distinguished by a high galacturonic acid content, with glucose as its major neutral sugar. LICP007-N1 also contained xylose and mannose, which were absent from the two acidic fractions. These results indicate that the xylose- and mannose-containing polysaccharides in the crude extract are readily eluted by the neutral eluent and are therefore enriched in the neutral fraction. The monosaccharide molar ratios of the neutral and acidic polysaccharides also differed, indicating that the polysaccharide composition is related to the type of eluent. In addition, LICP007-S2 contained glucuronic acid, while LICP007-S5 contained galacturonic acid, indicating that the composition of monosaccharides may also be related to the concentration of eluent. The uronic acid content increased with the NaCl concentration: the content in LICP007-S5 (27.5%), eluted with 0.5 mol/L NaCl, was much higher than that in LICP007-S2 (3.6%), eluted with 0.2 mol/L NaCl. Compared with LICP007-N1 and LICP007-S2, LICP007-S5 had better inhibitory activity against α-glucosidase and α-amylase, which may be related to its high content of galacturonic acid. Galacturonic-acid-rich pectic polysaccharides have been reported to show strong α-glucosidase inhibitory activity, possibly through interactions between carboxyl groups and the enzyme active site [24,25,26]; however, this proposed structure–activity relationship remains a hypothesis that requires further verification. Moreover, polysaccharides with higher glucose contents have been reported to show higher glycosidase inhibitory activity [27,28], which is consistent with our results. Together, these results showed clear differences in monosaccharide profiles between the neutral and acidic polysaccharides as well as between the two acidic polysaccharides.

#### 2.5.3. FT-IR Spectroscopy

**Figure 5:**
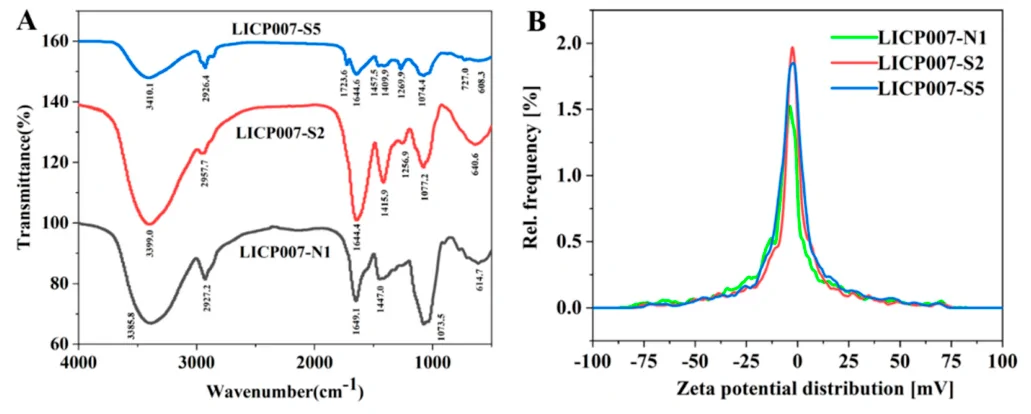
FT-IR spectra (**A**) and zeta potentials (**B**) of LICP007-N1, LICP007-S2, and LICP007-S5.

The FT-IR spectra of LICP007-N1, LICP007-S2 and LICP007-S5 in the range of 4000–400 cm^−1^ are shown in Figure 5A. All three spectra displayed the two characteristic absorption regions of polysaccharides: a strong and broad O–H stretching band between 3385.8 and 3410.1 cm^−1^ and a weaker C–H stretching band between 2926.4 and 2957.7 cm^−1^. The strong peaks at 1649.1 and 1447.0 cm^−1^ (LICP007-N1) and 1644.4 and 1415.9 cm^−1^ (LICP007-S2) were assigned to the asymmetric and symmetric stretching vibrations of the carboxylate groups of uronic acid. However, the absorption near 1649 cm^−1^ may also contain contributions from the amide I band of residual protein, so this assignment should be regarded as tentative [29,30]. In contrast, LICP007-S5 had a weak but clear peak at 1723.6 cm^−1^, indicating the presence of esterified (methyl-esterified) uronic acid groups [31,32]. Monosaccharide analysis showed that the GalA content of LICP007-S5 (27.5%) was much higher than the uronic acid contents of LICP007-N1 (GlcA, 1.6%) and LICP007-S2 (GlcA, 3.6%), consistent with the FT-IR results. The weak bands near 1256.9 cm^−1^ in both acidic fractions, together with those at 1415.9 cm^−1^ (LICP007-S2) and 1457.5 cm^−1^ (LICP007-S5), corresponded to symmetrical deformations and angle vibrations of C–OH groups. The absence of this absorption near 1200 cm^−1^ in LICP007-N1 may serve as a marker distinguishing the acidic polysaccharides (LICP007-S2 and LICP007-S5) from the neutral LICP007-N1 and may partly explain the differences in in vitro enzyme inhibitory activity among the three fractions. The absorption peaks in the region of 1200–1000 cm^−1^ were attributed to the stretching vibration of the pyranose ring [33]. LICP007-N1, LICP007-S2 and LICP007-S5 all had absorption peaks at approximately 1070 cm^−1^, which indicated the presence of pyranose rings in the three purified polysaccharides. The main FT-IR absorption bands and their assignments are summarized in Table 4.

#### 2.5.4. Zeta Potential

The surface charge of polysaccharides reflects the stability of their solutions or colloids and thus affects their potential applications. In this study, the zeta potentials of the three fractions were all slightly negative, ranging from −0.2 to −8.7 mV (Figure 5B), with LICP007-S5 showing the least negative value. The results showed that the three polysaccharides were in an unstable state in aqueous solution. LICP007-S5 and LICP007-N1 were also prone to coagulation, and the coagulation ability of LICP007-S5 was stronger than that of LICP007-N1.

#### 2.5.5. AFM Analysis

**Figure 6:**
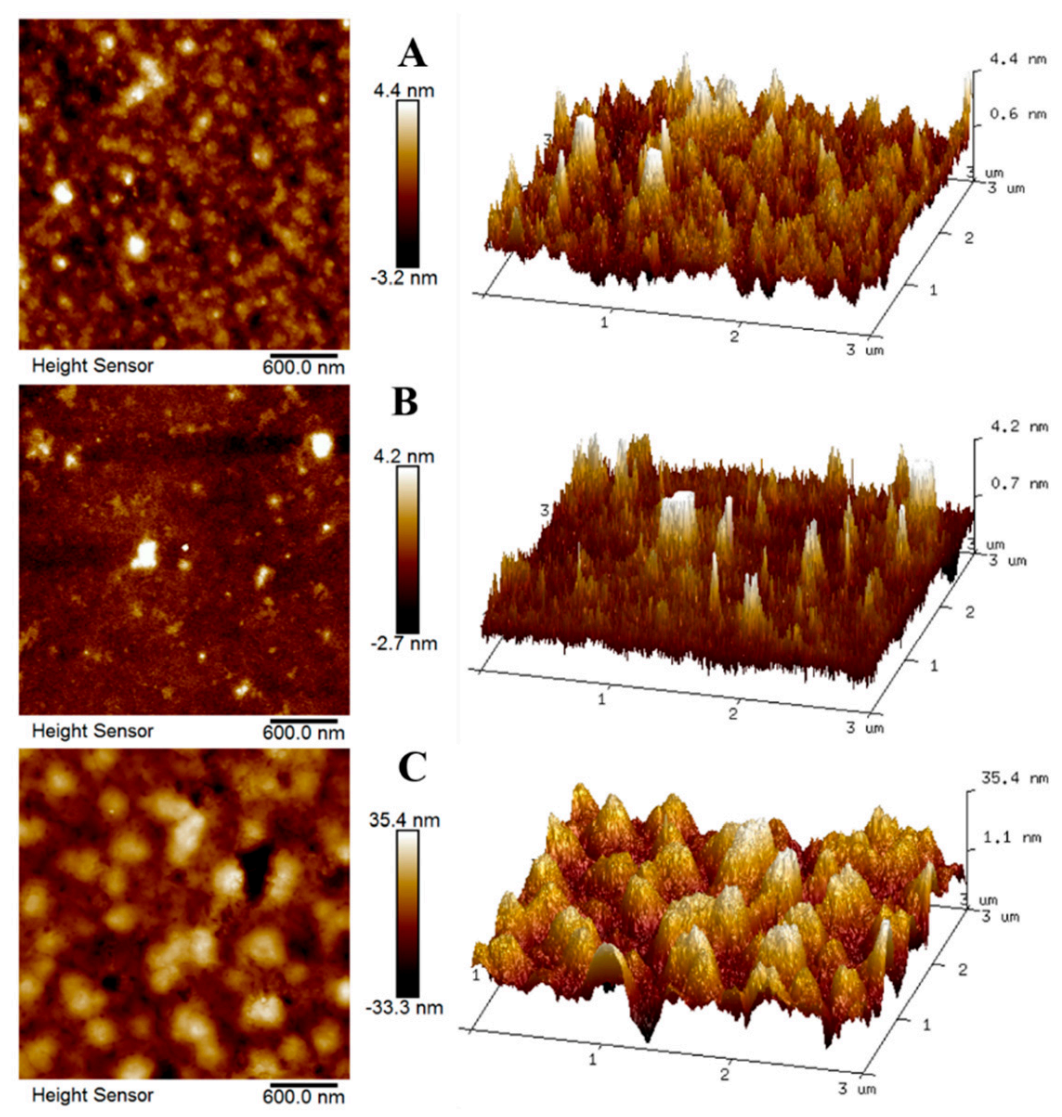
AFM images of LICP007-N1 (**A**), LICP007-S2 (**B**), and LICP007-S5 (**C**).

AFM is a powerful tool to observe the surface structure and roughness of polysaccharides with minimal sample preparation. The topographical AFM planar and 3-dimensional images of LICP007-N1, LICP007-S2 and LICP007-S5 are shown in Figure 6. LICP007-N1 showed flame-like aggregates of uniform size, with multiple closely arranged strands forming a uniform, compact, network-like micromorphology. Compared with LICP007-N1, the polysaccharide chain of LICP007-S2 was sparsely arranged, and the overall appearance seemed to be less compact but still showed a polysaccharide chain of flame-like aggregates with some irregular pores. In contrast, the polysaccharide chains of LICP007-S5 were arranged in short, stubby strands, and loose coacervates gathered and packed together to form large aggregates with denser and larger pores. In addition, the height of LICP007-S5 was approximately 35.4 nm, much higher than those of LICP007-N1 and LICP007-S2 (both approximately 4.4 nm), suggesting that LICP007-S5 may adopt an ordered, possibly triple-helical conformation; however, AFM height measurements alone cannot confirm helix formation [34,35]. Thus, clear differences in appearance and morphology existed between the neutral and acidic polysaccharides and between the acidic polysaccharides eluted at different NaCl concentrations, which may arise from their different uronic acid contents. These results indicated that monosaccharide composition and the cross-links between monosaccharide molecules could influence network structure formation.

#### 2.5.6. Congo Red Test

**Figure 7:**
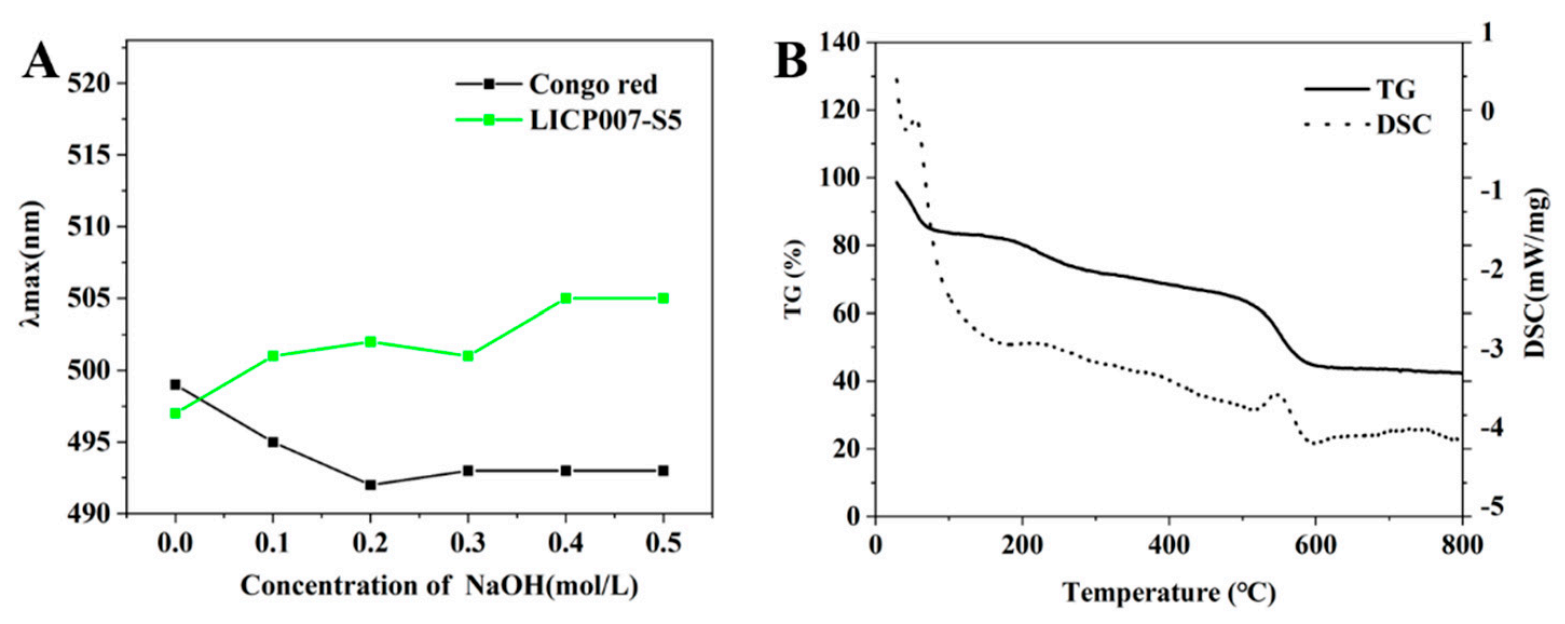
Congo red test (**A**) and TG–DSC curves (**B**) of LICP007-S5.

In general, a triple-helical polysaccharide interacts with Congo red, and the conformational transition induced by NaOH causes a bathochromic shift in the maximum absorption wavelength [36,37,38]. The conformation of LICP007-S5 was determined using the Congo red test. Figure 7A shows the change in the maximum absorption wavelength (λ_max_) of the LICP007-S5–Congo red complex in NaOH solutions (0–0.5 mol/L); a bathochromic shift in λ_max_ was observed at all NaOH concentrations, suggesting that LICP007-S5 may adopt an ordered, possibly triple-helical conformation; it should be noted that a bathochromic shift in the Congo red test provides indicative rather than conclusive evidence of a triple helix, and complementary techniques (e.g., circular dichroism) would be needed for definitive confirmation [34,37]. These results were consistent with the AFM observations.

#### 2.5.7. TG Analysis

TG analysis was used to evaluate the thermal stability of the polysaccharides, an important parameter for biological applications. Figure 7B shows the TG curve of LICP007-S5 from 35 to 800 °C. Below 100 °C, LICP007-S5 showed a weight loss of approximately 13%, attributed to the loss of absorbed water. Between 100 and 500 °C, the sample underwent a gradual weight loss of approximately 20%, which may correspond to the slow degradation of the polysaccharide matrix. The main weight loss occurred between 500 and 600 °C (approximately 22%), where LICP007-S5 underwent rapid thermal decomposition and its skeleton broke down. Above 600 °C, the weight remained almost unchanged, with a final residue of approximately 43% at 800 °C. These results indicate that LICP007-S5 exhibits a three-stage thermal degradation profile, with its major structural decomposition occurring between 500 and 600 °C.

#### 2.5.8. NMR Analysis

**Figure 8:**
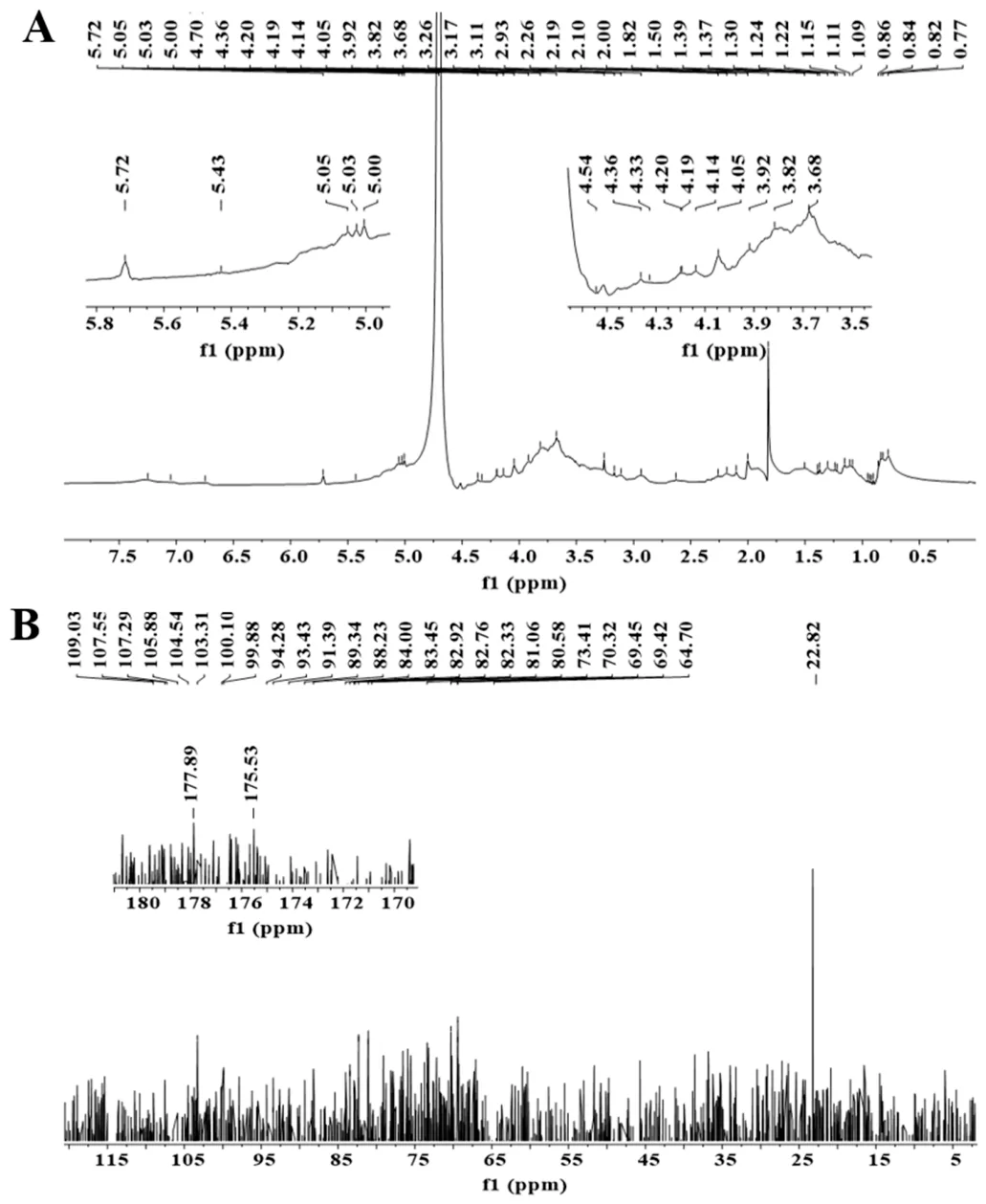
^1^H NMR (**A**) and ^13^C NMR (**B**) spectra of LICP007-S5.

NMR spectroscopy is a convenient technique for elucidation of the structural properties of polysaccharides. Both ^1^H and ^13^C NMR spectra of LICP007-S5 showed the typical characteristics of polysaccharides. As shown in Figure 8, the strong peak at 4.70 ppm could be attributed to the D_2_O solvent. Six obvious chemical shifts in anomeric protons at *δ* 5.43, 5.05, 5.03, 5.00, 4.54 and 4.36 ppm appeared both above and below δ 5.0 ppm, indicating the existence of both *α*- and *β*-configurations. The chemical shift appearing at approximately *δ* 5.65 ppm indicated that there was uronic acid in LICP007-S5. The anomeric proton signals at *δ* 5.43 ppm and *δ* 5.03 ppm were tentatively assigned to α-D-Glc*p*, and the signals at *δ* 5.05 ppm were tentatively assigned to the H-1 of α-1,5-linked Ara*f*. Meanwhile, the intense signal at 3.68 ppm was tentatively attributed to the methoxyl (O–CH_3_) groups of methyl-esterified galacturonic acid residues [32], and the signal at *δ* 1.82 ppm, together with the ^13^C signal at *δ* 22.82 ppm, was assigned to O-acetyl groups [39]. Similarly, the signals at *δ* 177.89 ppm and *δ* 175.53 ppm in ^13^C NMR could be attributed to carboxyl groups corresponding to the galacturonic acid units. The carbon signals appearing at *δ* 109.03 ppm and *δ* 107.55 ppm could be assigned to the C-1 of α-Ara*f* and 1,5-α-Ara*f*, and the signals at *δ* 99.88 ppm could be assigned to the C-1 of α-GalA*p*. The chemical shift appearing at *δ* 69.45 ppm indicated O-substituted C-6 of certain sugar residues. Moreover, the intense signal at *δ* 22.82 ppm suggested the presence of acetyl groups of the monosaccharide units. It should be noted that these assignments are based on 1D NMR spectra only and should be regarded as tentative. Due to severe signal overlap in the anomeric region, integration-based quantification of the individual anomeric signals was not performed; these assignments should be further verified by 2D NMR experiments (1H–1H COSY and HSQC) in future work.

## 3. Materials and Methods

### 3.1. Materials and Chemicals

Dried fruits of *L. barbarum* were obtained from Ningxia Zhongqi Wolfberry Trading Group Co., Ltd. (Zhongning, China) on 1 May 2017. Redistilled phenol, DC Protein Assay Kit, DEAE-52 cellulose, and Sephadex G-200 were purchased from Solarbio Science & Technology Co., Ltd. (Beijing, China); α-amylase, α-glucosidase, acarbose, soluble starch, and pNPG were purchased from Shanghai Source Leaf Biotechnology Co., Ltd. (Shanghai, China). All other chemicals were of analytical grade or higher. The following equipment was used: a UV spectrophotometer, a Biosafer-10B Vacuum Freeze Dryer (Biosafer, Nanjing, China), a KQ-250DE-type CNC ultrasonic washer (Kunshan Ultrasonic Instrument Co., Ltd., Kunshan, China), a BSA224S-CW electronic analytical balance (0.1 mg readability; Beijing Cedolis Instrument System Co., Ltd., Beijing, China), an Agilent 1260 liquid chromatograph (Agilent Technologies, Santa Clara, CA, USA), a 2200 evaporative light scattering detector, a DHG-9140A-type electric thermal constant temperature drum air drying oven (Shanghai Jinghong Experimental Equipment Co., Ltd., Shanghai, China), a Flying Pigeon Brand High-Speed Centrifuge (Shanghai Anting Scientific Instrument Factory, Shanghai, China), a Varioskan Flash enzyme marker (Thermo Scientific, Waltham, MA, USA), a porous hydroxymethacrylate polymer column (A4000) (300 mm L × 8.0 mm I.D., 10 μm) and an A7 guard (50 mm L × 8.0 mm I.D., 10 μm, made in Japan) were purchased from Malvern Panalytical Ltd. (Malvern, Worcestershire, UK), including an OMNISEC GPC/SEC system. A high-shear dispersing emulsification (HSDE) machine (IKA ULTRA TURRAX, T25 digital; IKA-Werke GmbH & Co. KG, Staufen, Germany) and Vivaflow^®^ 200 cross flow cassettes (Sartorius AG, Göttingen, Germany) were used.

### 3.2. Extraction and Separation of the L. barbarum Polysaccharide Fractions

The procedure used to fractionate the *L. barbarum* polysaccharides with in vitro α-amylase and α-glucosidase inhibitory activity is illustrated in Figure 1. Four *L. barbarum* polysaccharide fractions with different *M*_w_s (LICP007, LICP008, LICP009, and LICP010) were obtained using our previously reported method with slight modifications [19]. First, the *L. barbarum* powder (100 g) was extracted with water (1 L) at 60 °C for 30 min using an HSDE machine (IKA ULTRA TURRAX, T25 digital) at 15,000 rpm, and the mixture was then centrifuged at 8000 rpm for 15 min. The crude polysaccharide aqueous solution was collected for subsequent separation using membrane technology. In this process, suitable membrane materials and a suitable molecular weight cutoff (MWCO) were selected from four Vivaflow^®^ 200 cross flow cassettes (Sartorius, Germany) with specific MWCOs (30, 10, 5, and 2 kDa). The membrane separation procedure was as follows: 600 mL of polysaccharide extract was obtained using Vivaflow^®^ 200 with an MWCO of 30 kDa at 0.8 MPa and a flow rate of 150 mL/min, and the membrane area was 100 × 100 mm^2^. When the volume of retentate was 25 mL, 50 mL of distilled water was injected from the discharge pipe of Vivaflow^®^ 200. After separation, 600 mL of the polysaccharide extract was divided into retentate and permeate solutions; the retentate solution was lyophilized to obtain LICP007, and the permeate solution was subjected to further separation with an MWCO of 10 kDa to obtain LICP008. The separation procedure was similar to the above-described process. Finally, the four *L. barbarum* polysaccharide fractions with specific *M*_w_s (LICP007, LICP008, LICP009, and LICP010) were isolated and lyophilized.

### 3.3. Purification of the Polysaccharide Fraction LICP007

LICP007 was dissolved in hot water, and the protein was removed with Sevage reagent (chloroform: water-saturated n-butanol = 4:1, *v*/*v*) [40]. After treatment, the upper solution was collected, and NH_3_·H_2_O was added to adjust the pH to 9.0. H_2_O_2_ solution was then added with stirring in a 60 °C water bath to a final concentration of 10% (*v*/*v*), and decolorization proceeded at 60 °C for 30 min. The decolorized solution was dialyzed (MWCO 3000 Da) to remove residual hydrogen peroxide and other small molecules, concentrated under reduced pressure, and then lyophilized for 48 h. The purified polysaccharide was obtained and loaded onto a DEAE-52 cellulose column (5.5 cm × 60 cm), sequentially eluted at a flow rate of 1 mL/min with deionized water and 0.2 mol/L NaCl solution, followed by 0.5 mol/L NaCl solution [41]. The collected eluent was monitored using the phenol–sulfuric acid method, and UV detection was performed with an ultraviolet spectrophotometer at 490 nm [42]. Then, the distilled water fraction was concentrated and lyophilized to obtain neutral polysaccharide LICP007-N1, and the fractions eluted with 0.2 mol/L and 0.5 mol/L aqueous NaCl were dialyzed (MWCO 3000 Da), concentrated, and lyophilized to obtain the acidic polysaccharides LICP007-S2 and LICP007-S5. LICP007-N1, LICP007-S2, and LICP007-S5 were dissolved in 1 mL deionized water, loaded on a Sephadex G-200 column (5.5 cm × 60 cm), and eluted with deionized water at a flow rate of 1 mL/min. The eluent was monitored by the phenol–sulfuric acid method and analyzed by ultraviolet spectrophotometry at 490 nm [43]. The three purified polysaccharides retained the designations LICP007-N1, LICP007-S2 and LICP007-S5.

### 3.4. In Vitro α-Amylase and α-Glucosidase Inhibitory Activity Assays

#### 3.4.1. Determination of α-Amylase Inhibitory Activity

α-Amylase and α-glucosidase inhibitors play an important role in decreasing the release of glucose from carbohydrates and delaying carbohydrate absorption in humans [44]. The α-amylase inhibitory activities of four crude polysaccharides (LICP007, LICP008, LICP009, and LICP010) were determined using the method of Xu et al. with slight modifications [45]. Briefly, 45 μL of polysaccharide solution (1–50 mg/mL for the crude polysaccharides; 0.5–20 mg/mL for the purified fractions) was mixed with 15 μL of α-amylase (0.02 mg/mL) and incubated at 37 °C for 10 min. Then, 60 μL starch solution (0.08%) was added to the mixture and incubated at 37 °C for 15 min. Hydrochloric acid solution (60 μL, 1 mol/L) was added to terminate the reaction. Finally, 20 μL of iodine solution (0.01 mol/L) was added. Phosphate-buffered saline (PBS; 15 μL) replaced the α-amylase solution in the blank group, and deionized water (45 μL) replaced the sample solution in the control (no-inhibitor) group. Acarbose was used as the positive control [46]. The absorbance was then measured at 630 nm with a microplate reader. The three purified fractions LICP007-N1, LICP007-S2 and LICP007-S5 were analyzed using the same procedure. The inhibition ratio was calculated using Equation (1):

(1) $$\mathrm{Inhibition}\;\mathrm{rate}\;=\;(1-\frac{\mathrm{Asc}-\mathrm{Asb}}{\mathrm{Ac}-\mathrm{Ab}})\times 100\%$$

where *Ab* represents the absorbance of the blank group, *Ac* represents the absorbance of the control group, *Asc* represents the absorbance of the sample group, and *Asb* represents the absorbance of the sample blank group.

#### 3.4.2. Determination of α-Glucosidase Inhibitory Activity

The α-glucosidase inhibitory activities of four crude polysaccharides (LICP007, LICP008, LICP009 and LICP010) were determined using a previously reported method with slight modifications [43]. Briefly, 50 μL of polysaccharide solution (1–50 mg/mL for the crude polysaccharides; 0.5–20 mg/mL for the purified fractions) was mixed with α-glucosidase solution (4 U, 50 μL) and incubated at 37 °C for 15 min. Then, 50 μL of pNPG solution (1 mmol/L) was added as a substrate, and the mixture was incubated at 37 °C for 15 min. Finally, 50 μL of Na_2_CO_3_ solution (0.2 mol/L) was added to terminate the reaction, and the absorbance was measured at 405 nm. The three purified fractions (LICP007-N1, LICP007-S2 and LICP007-S5) were tested using the same procedure. Acarbose was used as the positive control. PBS replaced the α-glucosidase solution in the blank group, and deionized water replaced the sample solution in the control (no-inhibitor) group. The inhibition ratio was calculated using Equation (1).

### 3.5. Structural Characterization

#### 3.5.1. Qualitative Analysis

Fehling’s, biuret, iodine, and ferric chloride tests of the four crude polysaccharide fractions (LICP007–LICP010) were performed according to a literature report [47].

#### 3.5.2. Determination of Protein, Total Carbohydrate and Uronic Acid Contents

The total carbohydrate content of the crude polysaccharide fractions (LICP007–LICP010) was measured using the phenol–sulfuric acid assay with D-glucose as the standard. The protein content was determined using the DC protein assay kit, and the uronic acid content was determined using the carbazole-sulfuric acid method [48].

#### 3.5.3. Determination of Molecular Weight

The molecular weights (*M*_w_) of LICP007-N1, LICP007-S2, and LICP007-S5 were estimated using an OMNISEC GPC/SEC system (Malvern Panalytical Ltd., Malvern, Worcestershire, UK) equipped with a porous hydroxymethacrylate polymer column (A4000). Each sample (2.0 mg) was dissolved in 1 mL of Na_2_SO_4_ solution and filtered through a 0.45 μm membrane; the flow rate was 0.7 mL/min. Calibration curves were prepared using dextran standards.

#### 3.5.4. Monosaccharide Composition Analysis

The monosaccharide composition was analyzed using ion chromatography (ICS5000, Thermo Fisher Scientific, Waltham, MA, USA) with an electrochemical detector. Monosaccharide standards were used for calibration, and the concentrations were calculated from the corresponding peak areas and response factors. LICP007-N1, LICP007-S2 and LICP007-S5 (10 mg each) were dissolved in 3 mol/L trifluoroacetic acid (TFA, 10 mL) and hydrolyzed at 121 °C for 3 h in sealed test tubes. The hydrolysates were evaporated under N_2_ to remove TFA, and the residues were dissolved in 10 mL of distilled water and mixed by vortexing. Then, 100 μL of the resulting solution was mixed with 900 μL of deionized water and centrifuged at 12,000 rpm for 5 min to obtain the supernatant for IC analysis. A 5 μL aliquot of the supernatant was then injected for ion chromatography under the following conditions: a Dionex CarboPac^TM^ PA20 column (3 × 150 mm) at 30 °C; mobile phase A: H_2_O, B: 15 mM NaOH, and C: 15 mM NaOH with 100 mM NaOAc; flow rate: 0.3 mL/min [49].

#### 3.5.5. Fourier Transform Infrared (FT-IR) Spectroscopy

The Fourier transform infrared (FT-IR) spectra of LICP007-N1, LICP007-S2 and LICP007-S5 were collected using a Thermo Nicolet Nexus 870 ESP FT-IR spectrometer (SpectraLab Scientific Inc., Markham, ON, Canada). The sample (2 mg) was mixed with dried KBr powder at room temperature, pressed into a pellet and scanned in the range of 4000–400 cm^−1^.

#### 3.5.6. Zeta Potential Measurement

The zeta potentials of LICP007-N1, LICP007-S2 and LICP007-S5 were measured using a Particle Size Analyzer Litesizer 500 (Anton Paar GmbH, Graz, Austria). Briefly, the samples were dissolved in distilled water at a concentration of 1 mg/mL and analyzed at 25 °C [50]. The measurement was performed three times.

#### 3.5.7. Atomic Force Microscopy (AFM) Analysis

LICP007-N1 (1.0 mg) was dissolved in 100 μL of ultrapure water to obtain a final concentration of 10 mg/mL. Subsequently, the solution was dropped onto a freshly cleaved mica substrate and allowed to dry at room temperature overnight. All measurements were performed at ambient pressure and humidity using a Bruker atomic force microscope (Bruker Instruments, Inc., Billerica, MA, USA). LICP007-S2 and LICP007-S5 were prepared using the same procedure.

#### 3.5.8. Congo Red Test

LICP007-S5 (2.0 mg) was dissolved in 2.0 mL distilled water and mixed with 2.0 mL of Congo red solution (80 μmol/L) [51]. Then, NaOH solution (1 mol/L) was gradually added to the mixture to achieve final NaOH concentrations of 0.1, 0.2, 0.3, 0.4 and 0.5 mol/L. The maximum absorption wavelength was determined by UV–Vis spectrometry in the 200–800 nm range [52].

#### 3.5.9. Thermogravimetric (TG) Analysis

TG analysis of LICP007-S5 was performed in the temperature range of 35–800 °C with a heating rate of 10 °C/min using an STA449C thermogravimetric analyzer (NETZSCH-Gerätebau GmbH, Selb, Germany).

#### 3.5.10. Nuclear Magnetic Resonance (NMR) Analysis

LICP007-S5 (50 mg) was dissolved in 1.0 mL of D_2_O and lyophilized for deuterium exchange; this process was repeated three times [53]. ^1^H NMR and ^13^C NMR spectra were recorded on a Bruker Advance III HD 400 MHz spectrometer (Bruker Instruments, Inc., Billerica, MA, USA) at 25 °C.

### 3.6. Statistical Analysis

All experiments were performed in triplicate, and the data are expressed as the mean ± standard deviation. Statistical differences were tested using one-way analysis of variance (ANOVA) followed by Tukey’s honestly significant difference (HSD) test. Differences were considered statistically significant at *p* < 0.05. All statistical analyses were performed using SPSS 19.0 (IBM Corp., Armonk, NY, USA), and figures were prepared using Origin 9.1 (OriginLab Corp., Northampton, MA, USA). The half-maximal inhibitory concentrations (IC50) were calculated by nonlinear regression fitting of the concentration–inhibition curves using GraphPad Prism 8.0 (GraphPad Software, San Diego, CA, USA).

## 4. Conclusions

In this study, one neutral polysaccharide (LICP007-N1) and two acidic polysaccharides (LICP007-S2 and LICP007-S5) were obtained from *L. barbarum* by membrane separation, DEAE-52 cellulose anion-exchange chromatography and Sephadex G-200 gel-filtration chromatography. Characterization results indicated obvious differences among the three fractions in molecular weight, monosaccharide composition and physicochemical properties. LICP007-S5, an acidic polysaccharide rich in galacturonic acid (27.5 mol%), showed the strongest in vitro α-amylase and α-glucosidase inhibitory activity; AFM and Congo red results suggest that it may adopt an ordered, possibly triple-helical conformation, but this requires further verification. The present data support LICP007-S5 as a potential in vitro enzyme inhibitor rather than a proven hypoglycemic agent. However, the detailed structure–activity relationship of these polysaccharides remains unclear and needs to be further investigated by methylation analysis and two-dimensional NMR spectroscopy. Furthermore, the inhibitory activity reported in this study is based solely on in vitro enzyme inhibition assays; the actual antidiabetic potential of LICP007-S5 must be verified in cellular models and in vivo studies.

## Figures and Tables

**Table 1:** Qualitative analysis results and chemical composition of crude polysaccharide fractions.

Items	LICP007	LICP008	LICP009	LICP010
Iodine reaction	−	−	−	−
Fehling reagent reaction	−	−	−	−
Biuret reaction	+	+	+	+
Ferric chloride reaction	+	+	+	+
Relative yield (%)	4.10	2.74	2.25	2.16
Protein (%)	12.7	14.5	19.3	15.9
Total carbohydrate (%)	65.3	52.7	57.4	49.8
Uronic acid (%)	18.2	13.3	15.3	10.7

Note: “+” indicates a positive reaction; “−” indicates a negative reaction.

**Table 2:** Molecular weight parameters of the three polysaccharide fractions from *Lycium barbarum* L.

Items	LICP007-N1		LICP007-S2	LICP007-S5	
	Peak 1	Peak 2	Peak 1	Peak 1	Peak 2
*M*_n_ (g/mol)	3.211 × 10^3^	5.112 × 10^4^	8784	4.769 × 10^3^	7.871 × 10^4^
*M*_w_ (g/mol)	4.229 × 10^3^	1.042 × 10^5^	5.368 × 10^4^	9.202 × 10^3^	1.031 × 10^5^
*M*_z_ (g/mol)	4.839 × 10^3^	1.388 × 10^5^	9.259 × 10^4^	1.690 × 10^4^	2.101 × 10^5^
PDI (*M*_w_/*_M_*_n_)	1.317	2.309	6.111	2.002	1.31
*SV*_g_ (cm^3^/g)	12.4339	0.1443	0.2764	6.8907	0.2261
*R*_g_ (nm)	82.7	4.85	99.74	38.31	4.13
Calc. *dn/dc* (mL/g)	0.0995	0.0995	0.1217	0.1566	0.1566

**Table 3:** Monosaccharide composition of LICP007-N1, LICP007-S2, and LICP007-S5.

No.	Proportion of Monosaccharides (Mole %)								
	Rha	Ara	GlcN	Gal	Glc	Xyl	Man	GalA	GlcA
LICP007-N1	2.2	50.2	3.0	27.0	11.3	3.0	1.8	−	1.6
LICP007-S2	3.3	38.1	2.2	28.6	24.4	−	−	−	3.6
LICP007-S5	4.6	21.6	2.5	12.4	31.4	−	−	27.5	−

**Table 4:** Main FT-IR absorption bands and assignments of LICP007-N1, LICP007-S2 and LICP007-S5.

Assignment/Vibration	LICP007-N1 (cm^−1^)	LICP007-S2 (cm^−1^)	LICP007-S5 (cm^−1^)
O–H stretching vibration of hydroxyl groups	3385.8	3399.0	3410.1
C–H stretching vibration	2927.2	2957.7	2926.4
Asymmetric stretching of carboxylate groups (COO–) of uronic acid (may overlap with amide I of residual protein)	1649.1	1644.4	–
Symmetric stretching of carboxylate groups (COO–)	1447.0	1415.9	–
C=O stretching of esterified (methyl-esterified) uronic acid	–	–	1723.6
Symmetrical deformation and angle vibration of C–OH groups	–	1256.9, 1415.9	1256.9, 1457.5
Stretching vibration of the pyranose ring	ca. 1070	ca. 1070	ca. 1070

## Data Availability

The authors declare that all data supporting the findings of this study are available within the paper and any raw data can be obtained from the corresponding author upon request.
